# Allotransplanted Neurons Used to Repair Peripheral Nerve Injury Do Not Elicit Overt Immunogenicity

**DOI:** 10.1371/journal.pone.0031675

**Published:** 2012-02-09

**Authors:** Weimin Liu, Yi Ren, Adam Bossert, Xiaowei Wang, Samantha Dayawansa, Jing Tong, Xiaoshen He, Douglas H. Smith, Harris A. Gelbard, Jason H. Huang

**Affiliations:** 1 Department of Neurosurgery, University of Rochester, Rochester, New York, United States of America; 2 Center for Neural Development and Disease, University of Rochester, Rochester, New York, United States of America; 3 Department of Neurosurgery, Fourth Affiliated Hospital of Hebei Medical University, Shijiazhuang, Hebei Province, China; 4 Department of Neurosurgery, Xijing Hospital, Fourth Military University, Xi'an, China; 5 Center for Brain Injury and Repair, University of Pennsylvania, Philadelphia, Pennsylvania, United States of America; 6 Department of Neurology, University of Rochester, Rochester, New York, United States of America; University of North Dakota, United States of America

## Abstract

A major problem hindering the development of autograft alternatives for repairing peripheral nerve injuries is immunogenicity. We have previously shown successful regeneration in transected rat sciatic nerves using conduits filled with allogeneic dorsal root ganglion (DRG) cells without any immunosuppression. In this study, we re-examined the immunogenicity of our DRG neuron implanted conduits as a potential strategy to overcome transplant rejection. A biodegradable NeuraGen® tube was infused with pure DRG neurons or Schwann cells cultured from a rat strain differing from the host rats and used to repair 8 mm gaps in the sciatic nerve. We observed enhanced regeneration with allogeneic cells compared to empty conduits 16 weeks post-surgery, but morphological analyses suggest recovery comparable to the healthy nerves was not achieved. The degree of regeneration was indistinguishable between DRG and Schwann cell allografts although immunogenicity assessments revealed substantially increased presence of Interferon gamma (IFN-γ) in Schwann cell allografts compared to the DRG allografts by two weeks post-surgery. Macrophage infiltration of the regenerated nerve graft in the DRG group 16 weeks post-surgery was below the level of the empty conduit (0.56 fold change from NG; p<0.05) while the Schwann cell group revealed significantly higher counts (1.29 fold change from NG; p<0.001). Major histocompatibility complex I (MHC I) molecules were present in significantly increased levels in the DRG and Schwann cell allograft groups compared to the hollow NG conduit and the Sham healthy nerve. Our results confirmed previous studies that have reported Schwann cells as being immunogenic, likely due to MHC I expression. Nerve gap injuries are difficult to repair; our data suggest that DRG neurons are superior medium to implant inside conduit tubes due to reduced immunogenicity and represent a potential treatment strategy that could be preferable to the current gold standard of autologous nerve transplant.

## Introduction

Each year approximately 360,000 people in the United States suffer a peripheral nerve injury (PNI), which is a leading source of lifelong disability [1]. The most frequent cause of PNIs is motor vehicle accidents, while gunshot wounds, stabbings, and birth trauma are also common factors. Patients suffering from disabilities as a result of PNIs are also burdensome to the healthcare system, with average hospital stays of 28 days each year [1]. Currently, the most reliable choice in repair of major defects in peripheral nerves is autologous nerve grafts [2]. The introduction of an autologous axon segment provides a physical and biological scaffolding to promote axonal outgrowth. Previous work has shown that this technique appears to be the best option for bridging axotomies in clinical situations [3]. The autograft serves as a physical guide composed of morphologically native biomaterial, which allows for the progression of “sprouting” axons from the proximal end to the distal nerve stump. However, donor autologous nerves are obviously limited in availability and necessitate another surgical site with added potential for wound morbidity. Accordingly, in recent years there has been considerable interest in developing alternative strategies to repair damaged peripheral nerves through combinations of transplanted biological or synthetic materials [4], [5], [6], [7].

A combination approach of particular interest is using synthetic tubes seeded with Schwann cells to direct axon regeneration [4], [8], [9], [10], [11]. However, like most cells, Schwann cells express major histocompatibility complex class I (MHC I), which occupy the most gene-dense region of the mammalian genome and play an important role in the immune system and autoimmunity. Thus, there has been debate regarding the utility of allogeneic Schwann cells for nerve repair due to their capacity to elicit host immune attack. While the use of immunosuppressive therapy has been proposed to reduce this immune response, it introduces complications of its own.

In contrast to Schwann cells, neurons represent one of the few classes of cells that do not express MHC I. While there are exceptions, such as neurons with MHC I expression in response to functional impairment or traumatic injury [12], [13], [14], neurons in general may represent a relatively immune-privileged allogeneic cell source. For example, neurons from DRGs have been isolated from either fetal or adult animals and adult human organ donors, and appear to survive well in culture and following transplantation, which makes them candidates in finding the biologic alternative to autografts [15]. Indeed, we have recently demonstrated consistent long-term survival of transplanted mechanically elongated DRG neuron constructs despite the absence of immunosuppressive therapy [16]. However, specific immune response of the host animal was not evaluated.

In the present study, we assessed the immunogenicity of DRG neuron nerve grafts compared to Schwann cell transplantation. We repaired 8 mm sciatic nerve gaps using NeuraGen® nerve guides (NG; Integra Lifesciences Corp, Plainsboro, NJ, USA) seeded with either allogeneic DRG neurons or Schwann cells transplanted from one strain of rat (Wistar) to another (Sprague Dawley), and evaluated the regeneration of the sciatic nerve and immune response using ELISA and immunohistochemistry (IHC).

## Materials and Methods

### DRG isolation

All animal experiments were approved by the University of Rochester Animal Care Committee. The DRGs from C1 to L1 spinal segments of both sides were dissected from three-day-old pups of the Wistar (Harlan, Charles River, Wilmington, MA) rat strain after decapitated using standard techniques [16]. DRGs were dissected aseptically and collected in ice cold Lebovitz's L-15 non-CO_2_ sensitive balanced medium (Invitrogen, Carlsbad, CA).

### DRG cell culture

After trimming of connective tissue, fat and nerve roots, the DRGs were transferred into 0.1% collagenase (Sigma-Aldrich, St. Louis, MO) in Neurobasal A media (Invitrogen, Carlsbad, CA) and incubated for 0.5 hours at 37°C followed by a treatment in fresh 0.1% collagenase for an additional 1 hour. Ganglia were then gently washed twice in Ca-Mg-free Hank's Balanced Salt Solution (HBSS) (Invitrogen, Carlsbad, CA). The DRG pellets were resuspended in complete medium and mechanically separated using a fire polished pasture pipet until completely dissociated and passed through a pre-wetted 70 um nylon cell strainer (BD Falcon, Franklin Lakes, NJ). Cells were plated onto either slides or dishes previously coated with 0.1 mg/ml poly-L-lysine followed by 1.0 ug/ml laminin (Sigma-Aldrich, St. Louis, MO) and maintained in complete growth medium consisting of Neurobasal A Media supplemented with B27 (Invitrogen, Carlsbad, CA), 1 mM L-Glutamine (Invitrogen, Carlsbad, CA), 0.1 mg/ml penicillin/streptomycin (Invitrogen, Carlsbad, CA), and 50 ng/mL nerve growth factor 7S (Gibco Invitrogen, Carlsbad, CA). The 4-well chamber slides (BD Falcon, Franklin Lakes, NJ) were seeded at 5×10^4^ cells/well, the 35 mm dishes at 0.5×10^6^ cells/dish (Sigma-Aldrich, St. Louis, MO) and after cell attachment, DRG cultures were immediately treated with mitotic inhibitor cocktails consisting of 5 µM cytosine arabinoside, 20 µM 5-fluoro-2′-deoxyuridine (Sigma-Aldrich, St. Louis, MO) and 20 µM uridine (Sigma-Aldrich, St. Louis, MO).

### Schwann cell culture

Schwann cells were isolated using the same method as described in DRG isolation. The ganglia were digested in 0.25% trypsin (Invitrogen, Carlsbad, CA)/0.03% collagenase in HBSS and incubated three times at 37°C for 15 minutes. After trypsin inhibition and mechanical dissociation, the cell suspension was plated on poly-L-lysine and laminin-coated coverslips (15 mm) at 4×10^4^ cells/coverslip or 35 mm dishes at 0.5×10^6^ cells/dish in Dulbecco's Modified Eagle Medium/F12 (DMEM/F12, Gibco Invitrogen, Carlsbad, CA) supplemented with 10% fetal bovine serum (Sigma-Aldrich, St. Louis, MO), 1 mM L-Glutamine, 0.1 mg/ml penicillin/streptomycin, 10 ng/mL nerve growth factor 7S, and 1% N2 supplement (Invitrogen, Carlsbad, CA). During the first 24 hours (i.e. days in vitro 1:DIV1), 1% BSA (Sigma-Aldrich, St. Louis, MO) was added to the culture medium.

### Immunocytochemistry

After 1, 2, 3, 4 and 7 days of culturing, DRG or Schwann cells plated on chamber slides were washed with phosphate saline buffer (PBS), fixed with 4% paraformaldehyde in PBS for 30 minutes, and then rinsed with PBS. Cells were incubated with blocking solution (5% normal donkey serum in PBS with 0.1% Triton X-100) for 1 hour at room temperature, followed by 4°C overnight incubation with the primary antibodies MAP-2 (Chicken polyclonal; 1∶5000; Abcam, Cambridge, MA) and glial fibrillary acidic protein (GFAP, Rabbit affinity isolated; 1∶500; Sigma-Aldrich, St. Louis, MO). The secondary antibodies Alexa Fluor 488 goat anti-chicken (1∶500, Invitrogen, Carlsbad, CA) and Alexa Fluor 594 donkey anti-mouse (1∶1000, Invitrogen, Carlsbad, CA) were used to incubate cells for 2 hours at room temperature in the dark, which was followed by PBS washing. The slides were mounted with medium for fluorescence with DAPI (Vector Lab, Burlingame, CA) and examined using an Olympus Fluoview confocal microscope connected to a DP70 digital camera for bright phase, dark phase, and fluorescent image acquisition.

### Peripheral Nerve Injury (PNI) surgery and implantation of allogeneic cells

**Figure 1:**
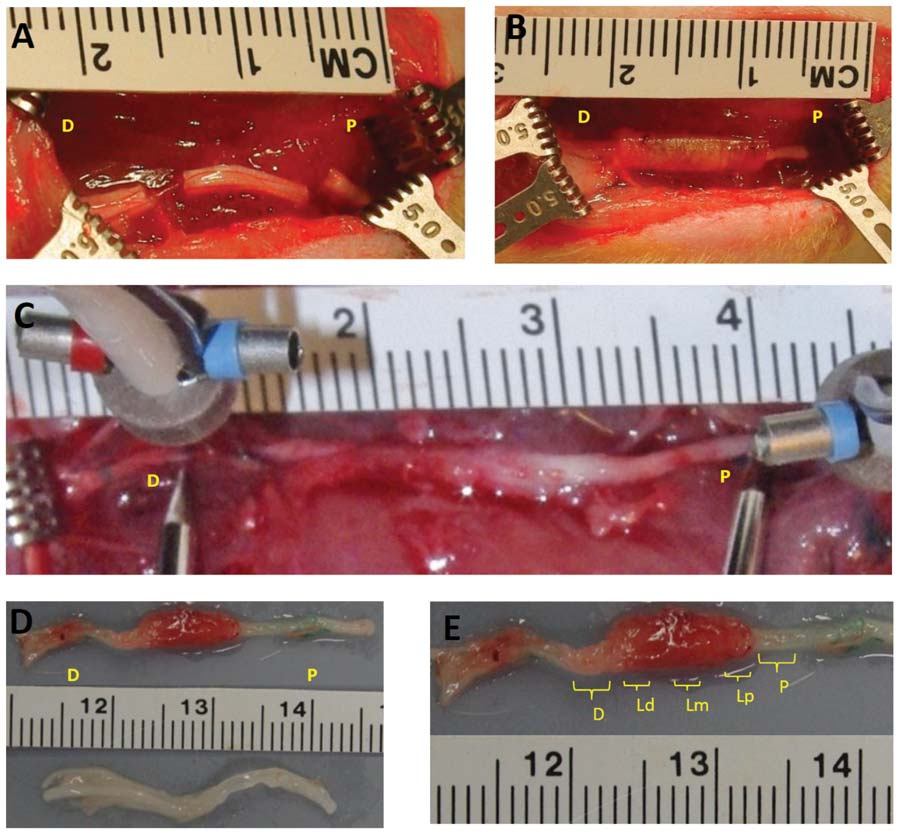
Surgical Procedures. (A) After skin incision, an 8 mm length of sciatic nerve was excised. (B) A NeuraGen® nerve guide tube of 2.0 mm ID×1.0 cm length was used to provide a physical guide for axons sprouting from the proximal nerve stump to reach the disconnected nerve segment. (C) The rats were sacrificed at 16 weeks post-PNI and sciatic nerves were harvested by excising 1.0 cm outside the NG tube. (D) The tube (top) is distinguishable from the contralateral healthy tissue (bottom) as an enlarged section of the nerve. The contralateral nerve served as a control for each rat. (E) For histological analysis, frozen sections were cut and examined at five locations: nerve proximal (P) and distal (D) to the tube and proximal (Lp), distal (Ld), and middle (Lm) longitudinal regions inside the tube.

Sciatic nerve injury was performed essentially as described by Huang and Smith [16]. All procedures were approved by the University of Rochester Animal Care Committee. Experimental subjects were adult male Sprague-Dawley rats (Harlan, Charles River, Wilmington, MA) (n = 24). The animals were divided into four groups: NeuraGen® nerve guide (NG; Integra Lifesciences Corp, Plainsboro, NJ) seeded with Schwann cells from Wistar rats (Wistar-Schwann, n = 6); NeuraGen® nerve guide seeded with DRG cells from Wistar rats (Wistar-DRG, n = 6); NeuraGen® nerve guide without cells (NG, n = 6); and Sham-operated control (Sham, n = 6). After buprenorphine analgesia and induction of anesthesia using Ketamine (100 mg/kg, I.P.), a skin incision was made in the left hind limb from the proximal region of the sciatic notch to the distal region of the popliteal fossa. The gluteal muscle was dissected to expose the sciatic nerve and its posterior tibial branch in order to excise an 8 mm length of sciatic nerve above its trifurcation (Fig. 1A). The NeuraGen® nerve guide tube (2.0 mm inside diameter (ID)×1.0 cm length) was used to provide a physical guide for axons sprouting from the proximal nerve stump to the disconnected distal nerve stump. For the animal groups receiving DRGs or Schwann cells, 1.5×10^6^ cells in 20 uL sterile PBS were placed in NG tubes using a syringe, making an allogeneic transplant that was sutured to the epineurium using four 8–0 nylon sutures (Fig. 1B). After nerve repair, the muscle and skin were closed with 6-0 vicry and 4-0 nylon sutures, respectively. The incision was closed, and the wound site was disinfected with Betadine (Purdue Products, Stamford, CT). Every two weeks, blood samples were collected under anesthesia using Ketamine (80 mg/kg, I.P) for ELISA data until 16 weeks after surgery when the animals were sacrificed.

### Enzyme-linked immunosorbent assay (ELISA)

Blood samples were collected from the tail vein 2, 4, 6, 8 and 16 weeks post-surgery. The cytokine levels in serum were measured by ELISA assay using a commercially available interferon (IFN)-γ ELISA kit (Invitrogen, Carlsbad, CA), as per instruction of the manufacturers.

### Histology for the sciatic nerves

16 weeks after surgery, rats were anesthetized using Ketamine/Xylazine and perfused intracardially using 1× PBS followed by 4% paraformaldehyde solution. Sciatic nerves were exposed and harvested 10 mm proximal and distal to the coaptation sites of the nerve guide constructs (Fig. 1C, D). Specimens were sequentially fixed in 15% sucrose/4% PFA at 4°C overnight and then dehydrated in 15% sucrose/1X PBS followed by 30% sucrose/1X PBS. 10–30 um thick frozen sections from the specimen were collected on a cryostat (Thermo Shandon Cryotome FSE Cryostats, Fisher Scientific, Pittsburgh, PA). For each excised nerve, axial slices were made beginning 2.0 mm from the proximal and distal ends of the construct while longitudinal slices were made along the length of the construct. Slices were stored at −80°C until use for histology. Each specimen was examined at five sites microscopically: nerve proximal (P) and distal (D) to the tube and proximal (Lp), distal (Ld), and middle (Lm) regions inside the tube (Fig. 1E).

### Immunohistochemistry

After rinsing in PBS, sections were incubated with blocking solution (5% normal donkey serum in PBS/0.1% Triton X-100) for 1 hour at room temperature. Slides were incubated with primary antibodies at 4°C overnight and with secondary antibodies at room temperature for two hours in the dark; all antibodies were diluted with 2% donkey serum in PBS/0.1% Triton X-100. The primary antibodies used are: Chicken anti-MAP2 (1∶2000, Abcam, Cambridge, MA), Rabbit anti-MBP (1∶200, Chemicon, Billerica, MA), Rabbit anti-Laminin (1∶1000, Abcam, Cambridge, MA), Rabbit anti-NF-200 (1∶250, Chemicon, Billerica, MA), Mouse anti-ED1 (1∶500, Abcam, Cambridge, MA), and Mouse anti-MHC class I [OX-18] (1∶200, Abcam, Cambridge, MA).The secondary antibodies used are Alexa Fluor 488 Donkey anti-Chicken (1∶500, Invitrogen, Carlsbad, CA), Alexa Fluor 594 Donkey anti-Rabbit (1∶1000, Invitrogen, Carlsbad, CA), Alexa Fluor 488 Donkey anti-Rabbit (1∶1000, Invitrogen, Carlsbad, CA), and Alexa Fluor 594 Donkey anti-Mouse (1∶1000, Invitrogen, Carlsbad, CA). Slides were rinsed with PBS after each incubation, mounted with Medium for Fluorescence with DAPI (Vector Lab, Burlingame, CA), and examined under a BX41 Olympus Fluoview confocal microscope connected to a DP70 digital camera.

### Statistical Analysis

All quantitative immune response analyses of ELISA, ED1 immunoreactive macrophage and axon density data are expressed as mean ± the standard error of the mean (SEM). All statistical tests used Prism software (GraphPad, San Diego, CA). P values <0.05 were considered significant. For the ELISA data, experimental points represent triplicates with a minimum of 5–6 animals per group. Statistical comparisons were made between different experimental groups using one-way repeated measures analysis of variance test (ANOVA), followed by Bonferroni's Multiple Comparison Test. The macrophages were counted from 20× images of 10 um tissue sections. Each 0.15 mm^2^ area at five different locations (P, D, Lp, Lm, Ld; Fig. 1E) that were identical for each slide was counted three times per rat. Three rats from each experimental group were randomly chosen for ED1 immunoreactive macrophage. NF-200 expression served to identify axonal regeneration by quantifying axon density. Three rats from each experimental group were randomly chosen for the axon density count. The density of axons was counted from 40× images of 10 um distal tissue sections. Each slide was counted four times per rat. The sum of distal axonal density 16 weeks after PNI was calculated for each rat and was expressed as standard optical density (SOD) to generate an estimate of the total number of regenerated axons in the sciatic nerve. Counts were performed using Image-Pro Plus 6.0 software (Media Cybernetics, Bethesda, MD). One-way ANOVA test was used to determine significant differences in macrophage and axon density count followed by Tukey and Bonferroni multiple comparison post tests.

## Results

### Evaluating the health of cultured neurons as implantation to repair the peripheral nerve injury

**Figure 2:**
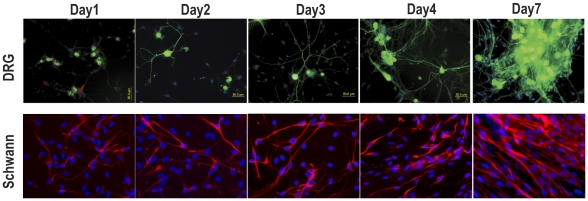
Evaluating the health of cultured neurons. (A) At the end of antimitotic treatment (day 3 and 4), the DRG cells (MAP-2 immunopositive neurons; green) had extended axons, and non-neuronal cells were essentially nonexistent. (B) Schwann cells demonstrated strong GFAP (red) expression and an elongated shape and dipolar morphology at day 2 and 3. 20×, Scale bar: 50 um. DAPI (blue).

To identify and assess the health of DRGs and Schwann cells, cultured cells were labeled with MAP2, a marker widely used to characterize neurons including DRGs, and with GFAP, a marker for Schwann cells. By DIV1, the DRG cells were firmly attached to the previously plated and extended neurites. DRG neurons were easily distinguished from Schwann cells by their distinctly larger diameter, phase-bright soma, and finer processes. At the end of the treatment with antimitotic agents (DIV3 and 4), the DRG cells had extended axons and the non-neuronal cells had essentially disappeared (Fig. 2A). By DIV2 and 3, Schwann cells showed healthy elongated shapes and dipolar morphologies (Fig. 2B). Based on these results, DRG or Schwann cells were injected into NG tubes and grafted into the animal groups receiving cells on culture DIV3–4 and 2–3, respectively.

### Recipient survival rate

All animals recovered well from the initial surgery for the duration of the 16 week *in vivo* study without signs of infection or immunorejection even though no immunosuppressive therapies were given. However, two animals (one from the Sham group, one from the Wistar-DRG group) died under general anaesthesia during blood sample collection and were therefore excluded from this study.

### Histological analysis of sciatic nerve regeneration

**Figure 3:**
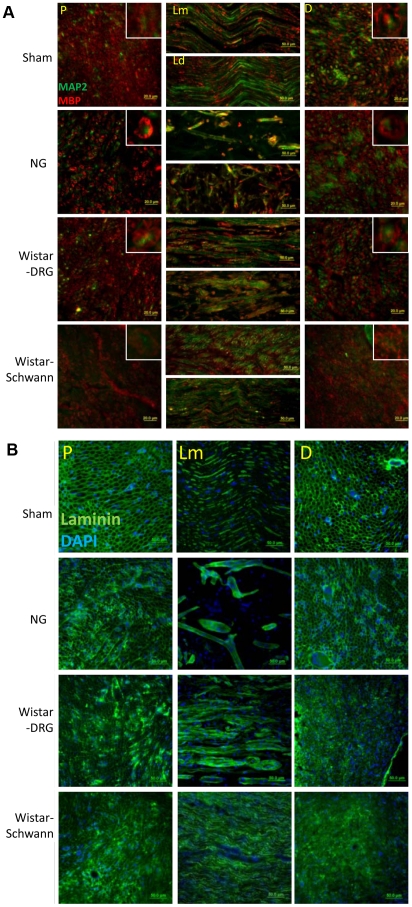
Histological analysis of the sciatic nerve 16 weeks post-PNI. For anatomical terminology, refer to *Fig. 1E*. (A) Myelination (MBP, green) of the regenerated axons (MAP2, red). Inset show the structure of individual myelinated axon fascicles, enlarged from 40× image. Axial sections 40×, longitudinal sections 20×. Thickness = 15 um. (B) Expression of laminin (green) was consistent with results from *Fig. 3A*. DAPI (blue), 20×. Thickness = 15 um.

16 weeks post-surgery, sciatic nerves were harvested and examined using histological methods. To examine the pattern of myelination of regenerated axons, we used MAP2 as a neuronal marker and MBP as a myelin maker. MAP2/MBP immunohistochemistry (Fig. 3A) demonstrated that the proximal and distal nerve stumps of the control animals exhibited axons surrounded by tightly packed and evenly distributed myelin rings. Throughout the inside of the conduit tube, the healthy Sham control group shows a wave pattern of axons and myelin. Individual axons were also myelinated when the transection was repaired using a hollow NG tube. However, the NG group exhibited significantly less myelin expression at the proximal stump compared to the Sham group while the axons appear in aggregates and myelin rings are more variable in size at the distal stump. The reduction in the number of axons in the NG group compared to the Sham group is also evident in the longitudinal sections, which show disconnected axons growing in random directions. The Wistar-DRG group exhibited similar structure and myelin distribution as the Sham group at the proximal stump, but loses the ring pattern at the distal stump such that many axons appear to be only partially myelinated. Myelination is enhanced within the conduit tube in the Wistar-DRG group and although both regions still appear disconnected, the middle region demonstrated the best connectivity and axon number compared to the distal region. The Wistar-Schwann group consisted of fewer and larger diameter myelin rings at the proximal stump compared to the control Sham group and significantly fewer and less distinct axons at both the proximal and distal stumps compared to all three groups. Similar to the Wistar DRG observations, the myelin at the distal stump in the Wistar-Schwann group appears fragmented but still surrounds the axons and has superior myelination compared to the empty NG tube. However, axons at the middle region of the tube, although more dense, appear as short strands growing in inconsistent directions. The distal site of the tube in the Wistar-Schwann group seems to recover the morphology most similar to that of the Sham despite more abundant axons. These observations suggested that DRG and Schwann cell-infused NG tubes are capable of enhancing myelination of regenerated axons, although recovery of the morphology comparable to a healthy sciatic nerve was not achieved within 16 weeks post-surgery.

Because laminin is a constituent of Schwann cell basal lamina and is known to be essential for Schwann cell differentiation and axon myelination [17], we also confirmed the myelination results with laminin immunohistochemistry (Fig. 3B). Similar to MBP expression, the Sham group shows a ring-patterned structure at the proximal and distal stumps and wave pattern inside the tube. The proximal and distal stumps for the NG groups were similar in appearance to the Sham. However, the mid-section of the tube revealed few thick and disconnected tube-like structures. Wistar-DRG group showed a pattern similar to the control at the proximal stump but rings of smaller diameter that are variable in size at the distal stump. Longitudinal sections revealed bundles of elongated parallel fibers, some of which appear disconnected. Wistar-Schwann group also shows healthy appearance at the proximal stump. The distal stump, however, retains very few distinguishable ring-like structures. In contrast, the mid-section of the tube has mostly recovered the appearance of a healthy nerve, although laminin expression was significantly more abundant compared to the Sham. Thus, the pattern of laminin was consistent with the previously observed pattern of myelination from MAP2/MBP IHC.

**Figure 4:**
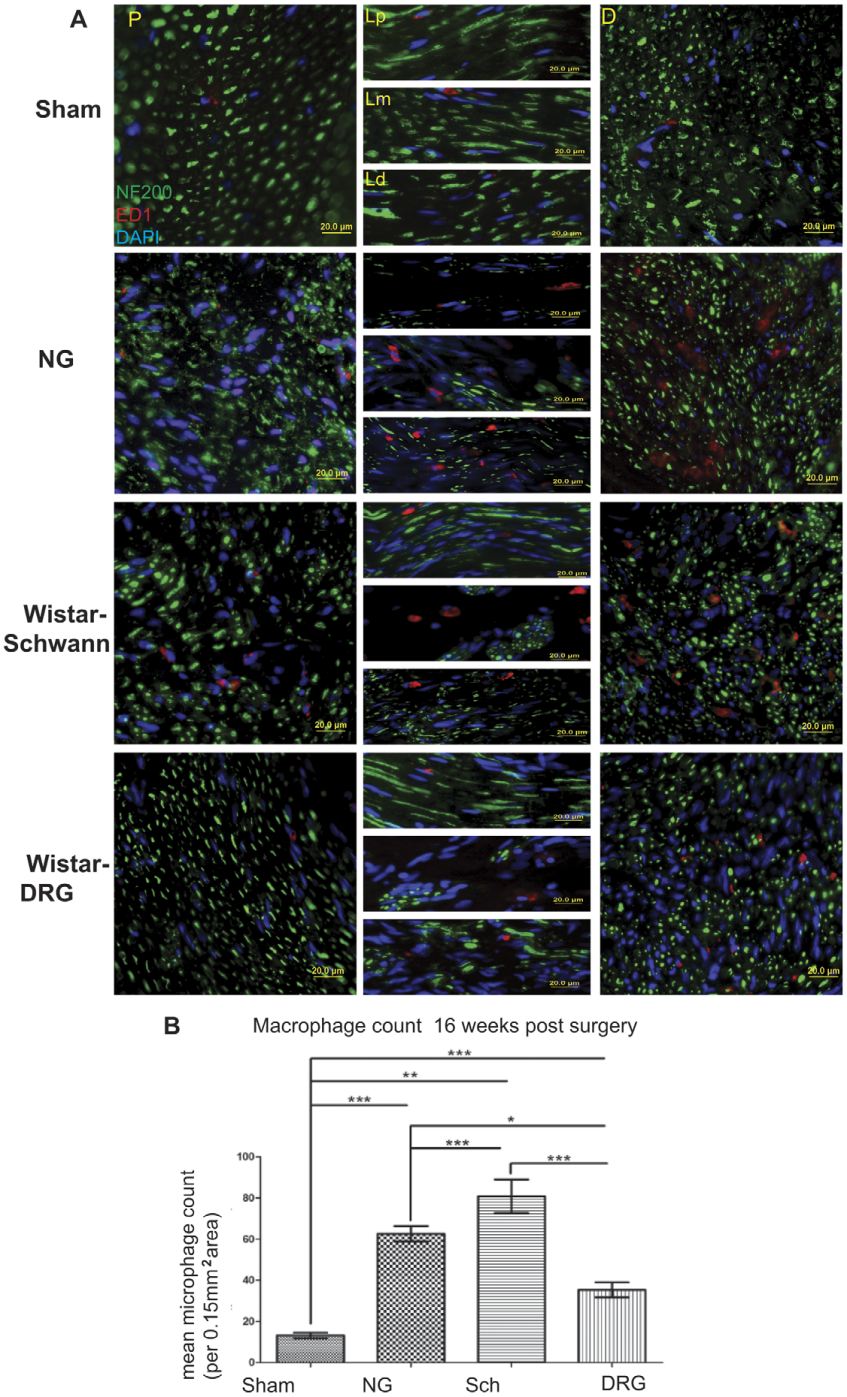
Histological and quantitative analyses of macrophage presence. For anatomical terminology, refer to *Fig. 1E*. (A) ED1 IHC shows macrophage (red) location and number in each group in neuronal structure (NF-200, green). Note Sham, Wistar-DRG, NG, and Wistar Schwann represent macrophage presence in increasing order in all tissue sections. DAPI (blue), 20×. Thickness = 15 um. (B) Quantification and statistical analysis of ED1 immunoreactive cells. Macrophage count reflects immunohistochemistry microscopy analysis in *Fig. 4A*. ANOVA Bonferroni and Tukey multiple comparison post tests determined significant differences in the mean macrophage count in each group: *P<0.05; **P<0.01; ***P<0.001. Both post tests found the same results. Error bars represent ± standard error of the mean.

**Figure 5:**
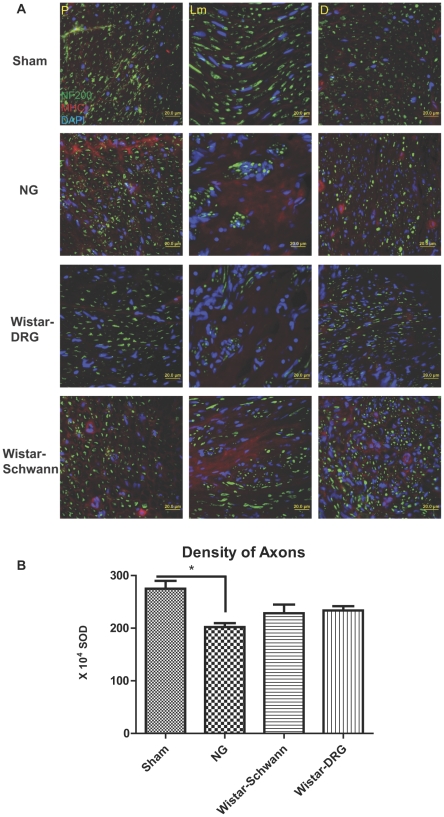
MHC I immunoreactivity (red) in neuronal structure (NF-200, green). For anatomical terminology, refer to *Fig. 1E*. (A) Increased MHC I immunoreactivity were observed in NG, DRG, and Schwann groups compared to the Sham group. DAPI (blue), 20×. Thickness = 15 um. (B) Axon regeneration estimated by NF-200 expressing axons. Axon density in distal stump was calculated and expressed as standard optical density (SOD) for each group. n = 3, 40× image of 10 um sections.

We next examined the immunohistochemical expression of neurofilament heavy intermediate filament protein (NF-200) and microphage infiltration using an antibody to the lysosomal membrane glycoprotein ED1 (Fig. 4), which is predominantly expressed in cells of mononuclear/macrophage lineage, and index of MHC I immunoreactivity in the regenerated sciatic nerve (Fig. 5A). NF-200 expression also served to identify axon morphology independent of myelination. Sham group exhibited evenly distributed axons at all sites inspected. The axon diameter seemed to be more variable in size in the distal stump compared to the proximal. Longitudinal sections revealed elongated fibers. The hollow NG graft significantly reduced the number of axons in the longitudinal sections, which appear as short strands rather than elongated fibers. Axons within the proximal stump were dispersed but not as evenly distributed as in a healthy nerve. The distal stump appeared to contain less axons compared to the Sham. The axon morphology at the proximal stump of the Wistar-DRG group appeared to resemble the Sham. However, axons in the distal stump were more abundant compared to the NG group but reduced in diameter compared to the Sham. Differences were observed among the three sites examined inside the tube: the proximal region contained elongated fibers and appeared healthy; middle region lacked elongated fibers but showed few aggregates of axons; and the distal region appeared to contain a mixture of aggregates and elongated fibers. Similarly, the Wistar-Schwann group exhibited a healthy morphology at the proximal region, aggregated of axons in the middle, and even fewer elongated fibers at the distal region within the tube. The proximal stump of the Wistar-Schwann group contained axons of larger diameter but uneven distribution. The distal stump appeared similar to the Wistar-DRG group. Thus, the distal stumps of all repaired nerves shared a similar pathological appearance while recovery of axon morphology at the proximal stump and inside the nerve guide was enhanced in Wistar-DRG and Wistar-Schwann groups.

To quantitatively assess axon regeneration, we counted the density of axons in the distal stump expressing NF-200 and compared among the groups (Fig. 5B). Compared to the Sham group, the NG group had significantly fewer axons at the distal stump, suggesting limited regeneration following the injury. In contrast, the Wistar-DRG and Wistar-Schwann groups did not significantly differ in axon density from the Sham group, indicating that the number of axons has almost recovered to similar levels as the healthy nerves in the Sham group. Although axon density in the DRG and Schwann allograft groups were not significantly higher than that of the NG group, the presence of DRG neurons and Schwann cells is correlated with an increasing trend in axon density and regeneration.

### Acute rejection assessment by serum levels of IFN-γ, quantification of macrophages, and immunoreactivity of MHC I

**Figure 6:**
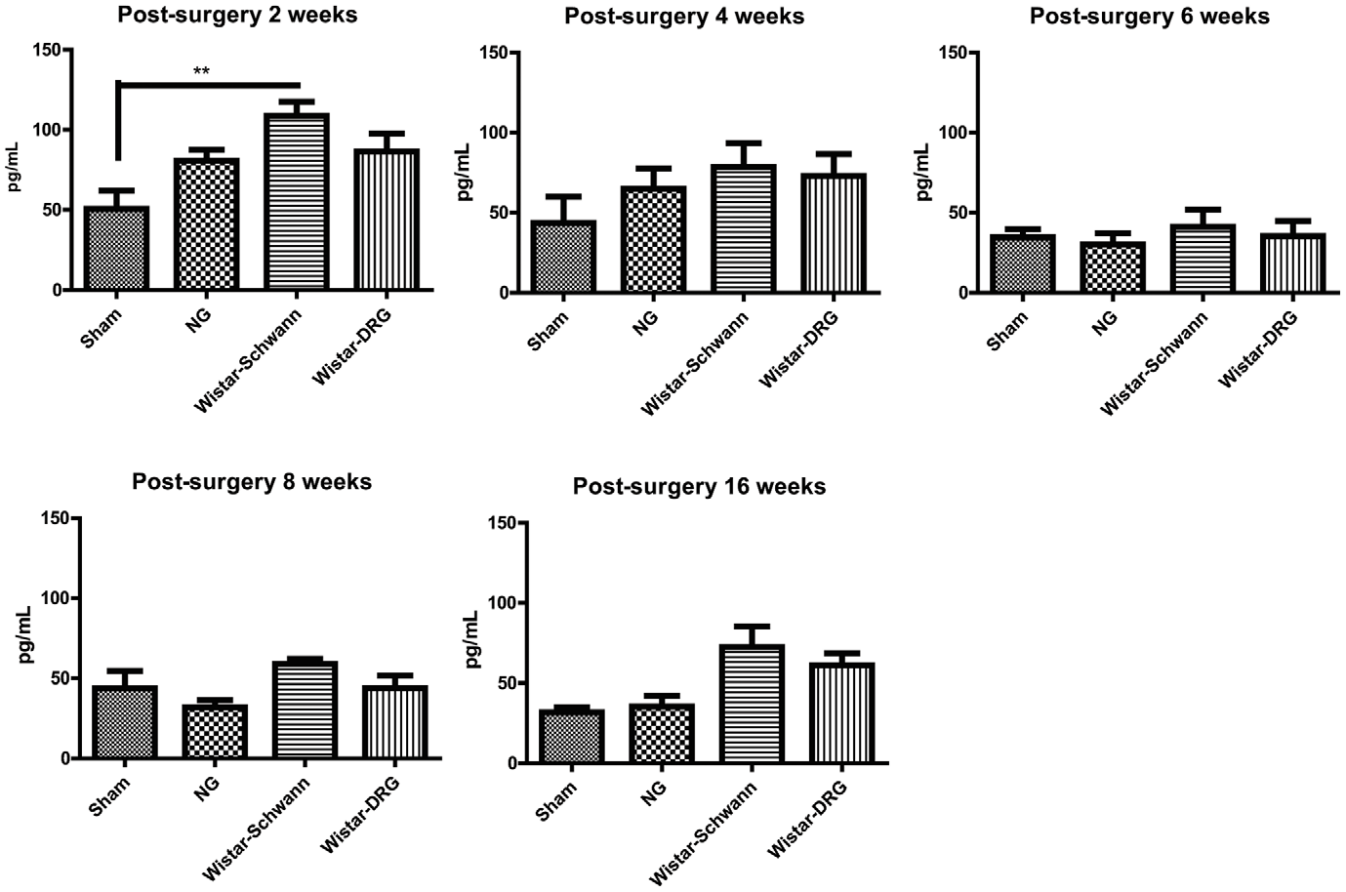
ELISA assays of IFN-γ in serum 2, 4, 6, 8 and 16 weeks post-surgery, respectively. IFN-γ levels of the allograft Wistar-Schwann group was higher than the Sham group in serum 2 weeks after surgery (P<0.01). For the NG and Wistar-DRG groups, IFN-γ concentrations in serum were not significantly different compared with that in sham control at all time points following PNI surgery. All measurements were done in triplicates. Data are representative of using n = 5, 6, 6, 5 mice in Sham, NG, Wistar-Schwann and Wistar-DRG groups, respectively.

Serum level of IFN-γ was determined by ELISA in all experimental groups 2, 4, 6, 8 and 16 weeks post-surgery. The IFN-γ level in the Wistar-Schwann group was higher than the Sham group 2 weeks after surgery (P<0.01). For the NG and Wistar-DRG groups, IFN-γ serum concentrations were not significantly different compared with the Sham control levels at all time points following PNI surgery (Fig. 6).

Anti-ED1 immunohistochemistry was used to determine macrophage count for each experimental group. The ANOVA test performed on macrophage count per group yielded strong evidence (P<0.0001) that the mean macrophage count for all experimental groups is significantly different. Both Bonferroni and Tukey multiple comparison post tests found the same significant differences between all groups (Fig. 4B). All operated groups had significantly elevated macrophage count from the Sham control group. The Wistar-DRG group was significant at the p<0.01 significance level whereas the Wistar-Schwann group was significant at the p<0.001 level. In addition, the Wistar-Schwann group was significantly elevated compared to the Wistar-DRG group at the p<0.001 level. These statistically significant findings can be visually confirmed using the immunohistochemistry data (Fig. 4A).

Major histocompatibility complex class I molecules (MHC I) are believed to be involved with direct allograft recognition and rejection by the host. These molecules are upregulated in response to injury [12] and are another measurement of immunogenicity and the host response to the varying material seeded within the nerve guides. Both the Wistar-Schwann and Wistar-DRG groups showed much higher MHC I expression when compared to the Sham and NG control groups (Fig. 5A). The pattern of MHC I expression coincided with that of laminin and MBP, primarily residing around rather than within the nerve fascicles. Compared to the Wistar-Schwann group, the Wistar-DRG group showed expression more confined to the region within the tube and at reduced levels at the proximal and distal stumps.

## Discussion

Many studies have attempted to repair PNIs using tissue engineered nerve conduits. Conduits have been seeded with various supporting cell types, secreted factors, and extracellular matrix proteins to assist their mechanical guidance [15]. Although cellular grafts have been shown to enhance regeneration compared to acellular grafts, a major problem is the host immune response associated with the cellular components [18]. Schwann cells are one of the supporting cell types that have received much attention for their potential to enhance regeneration of injured peripheral nerves [4], [8], [9], [10], [18]. Attempts have been made to overcome immunogenicity by using syngeneic Schwann cells [8], but this method is limited by the availability of compatible donors and may not improve morphological recovery above the capacity an acellular guide offers [4]. In this study, we were able to demonstrate successful regeneration of the transected sciatic nerve using nerve guides seeded with allogeneic DRG neurons and Schwann cells in the absence of immunosuppressant therapy.

By measuring IFN-γ, a pro-inflammatory cytokine, and macrophage count in our repaired nerve lesions, we are able to estimate the magnitude of the immune response that occurred in our experimental paradigms. Macrophages are key antigen presenting cells and recognize the MHC I molecules on foreign cells within the allograft. The macrophages then internalize the foreign cells, expose foreign antigen on their surface, and therefore help activate T helper (Th) cells which recruit stronger immune responses [19]. The Th cells secrete cytokines, such as IFN-γ, which is the most potent inducer of MHC I molecules and is also upregulated in the central nervous system (CNS) after traumatic injury [13], [20]. It is also present at elevated levels during the course of autoimmune diseases, such as multiple sclerosis, and in chronic neurodegenerative diseases [21].

In addition, Th cells can be regulated by IFN-γ. Expression of IFN-γ was found to regulate Th1 (activated) and Th2 (inactivated) expression by inhibiting Th2 [22]. Th1 response is associated with transplant rejection, while a Th2 response may contribute to tolerance and stable graft survival [23]. Therefore, as more Th cells are activated by macrophages, more Th1 cells are formed, which release IFN-γ, which propagates the immunorejection cascade. This process involving increased IFN-γ levels during rejection was also observed in heart and kidney transplants [24]. It is reported that Th1 polarization is related to the acute rejection in allograft transplants. Other cytokines including IL-2, IL-10, and INF-γ, can all accelerate T-cell mediated immune response while IL-4, 5, and 6 are helpful for B-cell mediated humoral immunity. Therefore, increases in IFN-γ during the course of our experiment can be correlated with immunorejection of the implanted conduit tubes.

The ELISA analyses demonstrated that heightened acute immune system activity was only observed in the Wistar-Schwann group. There were statistically significant increases in IFN-γ level by two weeks post-surgery for this group, likely corresponding to the end of the acute phase of PNI. Thus, allogeneic supporting Schwann cells seem to induce the upregulation of inflammatory cytokines such as IFN-γ, which are known to participate in immunorejection. This significant difference in upregulation was evident even at two weeks post-surgery, raising the possibility that more severe immune response could have taken place at earlier time points after PNI. In addition, the lack of significant differences at later time points may be because the assay was performed using plasma rather than tissue samples, which could have diluted the localized changes at the injury site. Thus, confirming the significance of IFN-γ upregulation by Schwann and other cell types may require further analysis at earlier time points after surgery by directly examining tissue samples near the injury site.

The ED1 immunoreactive cell count is another measurement of how the rats' immune systems responded to the interventions associated with each experimental group during long-term recovery. Since IFN-γ is important in recruiting macrophages [25] we would expect increased macrophage count where we observed increased IFN-γ levels in our ELISA analysis. We observed this pattern to a much greater degree than expected, having significant differences in all pairings of the experimental groups even at 16 weeks post-surgery, long after when the maximal immune response would have occurred. Next to the Sham control group, the Wistar-DRG group had the lowest macrophage count whereas the Wistar-Schwann group had the highest. These results were consistent with our IFN-γ findings that, there were significant differences that are suggestive of immunorejection long past the acute phase, further indicating that there may be intrinsic differences in the potential of DRG neurons and Schwann cells to elicit immune responses. We hypothesize that this observation is due to more abundant MHC I molecule presence in the Schwann cell allografts and reduced presence in the DRG cell allografts. In 1915, when allograft rejection was first suggested to be the result of test subject immune system response, the biologic mechanism was unknown [19]. It is now agreed upon that immunorejection occurs because allografts are foreign to the host. The idea that MHC antigen expressing cells, such as Schwann cells, are the immunogenic components that cause this conduit rejection has been proposed before [26]. We are also attributing our results to the acute immune response involved with MHC antigens and the resulting lymphocyte immune response described by Pattison et al [27].

MHC I expression and regulation is crucial for the immune response process, nerve regeneration, and functional recovery. Currently, MHC I molecules are believed to be involved with direct allograft recognition and rejection by the host. These molecules help direct the immune response by detecting unrecognized peptide sequences on foreign cells. This has been determined to be the case both in humans and other vertebrate animals [28]. MHC I molecules are vigorously upregulated immediately after peripheral axotomy when foreign cells are present and microglia are activated. Therefore, drastic nerve repair differences can result if MHC I levels vary among our experimental groups. MHC I mRNA have been shown to be constitutively expressed in peripheral motor neurons [13]. In particular, they are localized to motor axons and axon terminals and upregulated in the sciatic nerve following axotomy [12], [14]. We also demonstrated increased MHC I in all of our operated experimental groups compared to the healthy control (Fig. 5A). Besides mediating immune response, MHC I also appears to play an important role in nerve regeneration. In axotomized rat facial nerve, the expression of MHC I and II was detectable in the motor neurons 2–4 days post-operation [20]. The expression subsided after 12–18 days in crushed nerves, when the nerve had reinnervated the target muscle, but remained elevated in transected nerves. These observations demonstrate a correlation between MHC I expression and the degree of regeneration, which suggests that the relatively reduced level of MHC I in our Wistar-DRG group may represent a more advanced stage of recovery. Additionally, mice with genetically enhanced neuronal MHC I expression exhibited improved recovery of locomotor functions following spinal cord injury compared to wild-type mice [29]. In contrast, mice deficient in MHC I expression exhibited abnormal organization of the neuromuscular junction following regeneration and delayed motor function recovery [14]. This was attributed to impaired interaction between the axons and Schwann cells, resulting in inappropriate regulation of synapse formation. This shows that MHC immunoreactivity seems to have beneficial effects on promoting and sustaining axonal regeneration. Consequently, MHC I incompatibility between the allogeneic Schwann cells and the regenerating axons in the host rat in our experiments could have deleterious effects on regeneration of the sciatic nerve in addition to the inflammatory response.

A previous study comparing syngeneic and allogeneic Schwann cell transplantation revealed that allogeneic Schwann cells were rejected by 6 weeks and that the levels of MHC I and II expression increased between 2 and 3 weeks post-operation when using allogeneic Schwann cells whereas the levels decreased when syngeneic Schwann cells were used [11]. Thus, even if no significant differences in regenerative capacity could be identified, the use of Schwann cells to repair peripheral nerve lesions remains challenging due to its immunogenicity effects.

In contrast, DRG neurons possess unique immunological properties. The CNS is found to express reduced levels of MHC antigens and only expresses MHC II after injury whereas MHC I and II are expressed constitutively in the PNS [30]. These differences are likely the underlying reason for the immune-privileged state of the CNS and the differential response of the CNS and PNS to nerve injury, such as more rapid elimination of activated T cells in the CNS. While some studies have reported untreated embryonic DRG neurons to express MHC I *in vitro* [31], many *in vivo* studies suggest that the expression level is undetectable in healthy tissue and the upregulation following injury is primarily due to surrounding non-neural cell types [11].

While it is more likely that reduced DRG MHC I expression or host MHC I compatibility privileged the Wistar-DRG group with less immunogenicity, it is also possible that the implanted DRG cells slowed the degradation of the conduit tube, preventing the tube from eliciting a strong immune response. Studies have shown that biodegradable materials that the body absorbs more slowly elicit less foreign body immune response involving macrophages [5]. This was a proposed reason for immunogenic response differences observed among conduit tubes in Ghazanvi et al [6].

Previous studies have shown that allogeneic grafted tissue may elicit up to ten-times more immune response and MHC expression compared to autografts [32]. Our study used biodegradable, synthetic conduit tubes with allografted cells and demonstrated a reduced immune response in our DRG allografts compared to both the Wistar-Schwann and empty NG groups, suggesting that DRG allografts are capable of reducing the immune response compared to acellular conduits. Whether this reduction in immune response by using DRG allografts can achieve the same levels as autologous or syngeneic grafts will be the aim of our next study. In contrast, conduits filled with Wistar-Schwann cells exhibited high immune activity. This is expected because Schwann cells are credited as being the main antigen expressing immunogenic entity in nerve allografts [26].

Compared to the Wistar-Schwann group, the empty NG group was found to have less immunorejection, but axon regeneration was compromised. This tradeoff makes it difficult to determine which is a better repair method in the long run. Wistar-DRG appears to trump both problems by having a low immune response in addition to superior regeneration capabilities compared to the empty NG tube.

Both Schwann and DRG cells seemed to affect the morphology of the regenerating axon and the pattern of myelination at different sites within the injured nerve. A previous study has thoroughly examined the process of nerve regeneration in the rat up to 21 months post implantation and revealed that elongation and branching of axonal sprouts takes place within weeks while increases in the regenerated axon diameter only occurs several months after the repair [33]. This temporal dissociation is an efficient mechanism to prevent degeneration of the distal stump by ensuring initial reinnervation and subsequently refining the appropriate connections by eliminating extraneous axon sprouts. Thus, the differences in axon number observed in our IHC results may represent different stages of regeneration. The more abundant axons in the Wistar-Schwann group may reflect an earlier stage of regeneration during axonal sprouting while the Wistar-DRG group may have progressed to a later stage at which axonal refinement has occurred. In addition, the morphologic assessments may represent a mixed population of mature, immature, and degenerating axons since degeneration of regenerated axons and secondary demyelination may occur following nerve graft implantation [7], and the surviving axons may not have completely matured at 16 weeks post implantation. Thus, conclusive differences in morphologic recovery between the Wistar-DRG and Wistar-Schwann groups could not be observed using IHC and microscopy methods. Quantitative difference in axon regeneration was also not found by counting the density of axons at the distal stump of each group. These results suggest that, although DRG and Schwann cell transplantation may be more preferable for potentiating axon regeneration compared to an empty conduit, no significant difference in superior axon regeneration was found between the Wistar-DRG and Wistar-Schwann groups.

Furthermore, peripheral nerve regeneration is an interdependent process that requires interaction between axons and Schwann cells. While mature Schwann cells can survive in the absence of neurons, nerve injury can trigger de-differentiation into immature cells [10]. The immature cell type proliferates and, if remain deinnervated for an extend period of time, will not survive. Conversely, the nerve stump that results from nerve injury requires various factors released by Schwann cells in order to guide their growth [10]. Thus, an imbalance between the DRG and Schwann cells due to transplantation of exogenous cells may interfere with effective signaling between the two cell types and may not significantly enhance nerve regeneration. Indeed, Inhibitory effects of Schwann cells have been previously reported when seeded onto nerve guide grafts in similar attempts to repair transected rat sciatic nerves, including reduction in the number and diameter of regenerated axons and the proportion of myelinated axons [9].

Although many studies have considered Schwann cell transplantation as means to repair injured peripheral nerves due to its various roles in enhancing axonal growth, DRG cell implantation may be an equally reasonable option due to its mitogenic effects on Schwann cells [17]. In fact, implantation of DRG neurons may be more desirable because they can stimulate the proliferation and migration of endogenous Schwann cells and accelerate axon growth without the concern of immunogenicity. DRG and Schwann cells both have the potential to enhance axon regeneration, but may act through different mechanisms, which likely resulted in the minor morphological differences observed between the Wistar-DRG and Wistar-Schwann groups. Furthermore, the axons from the transplanted DRG neurons, which are lacking in Schwann cells, could also have contributed to improved regeneration in the Wistar-DRG group. Satellite cells are also likely to be present in the DRG neuron cultures, which could secrete tropic factors or other molecules and affect neurite outgrowth. Whether these contributions are significant for morphological and functional recovery will be examined in future studies.

Our results suggest that Schwann cell-mediated immunity may be associated with, and a part of, the transplant rejection response, while pure DRG neurons may contribute to tolerance and stable graft survival. In comparison to Schwann cells, conduit tubes infused with cultured allogeneic DRG cells present a promising alternative method for repairing nerve gaps in injured peripheral nerves by substantially reducing the possibility of rejection without compromising regenerative capabilities. By successfully finding a method that reduces the host immune response to foreign implanted cells during nerve repair, our study helps to establish a promising future for allografted conduit nerve repair that could be applied to long nerve lesions. While the functional motor and sensory recovery using this method needs to be verified in future studies, replacing the current “gold standard” of repairing nerve lesions using autografts with nerve guides that do not sacrifice healthy motor or sensory functions is becoming more of a reality each day.
